# Does Surgeon Specialty Make a Difference in Ventral Hernia Repair With the Component Separation Technique?

**DOI:** 10.7759/cureus.26290

**Published:** 2022-06-24

**Authors:** Mark M Yazid, Alexa De la Fuente Hagopian, Souha Farhat, Andres F Doval, Anthony Echo, Kevin Y Pei

**Affiliations:** 1 Institute for Reconstructive Surgery, Houston Methodist Hospital, Houston, USA; 2 General Surgery, Parkview Health, Fort Wayne, USA

**Keywords:** component separation techniques, clinical care outcomes, plastic surgery, general surgery, nsqip, incisional hernia

## Abstract

Background

Abdominal wall reconstruction (AWR) has evolved with the continued advent of new techniques such as component separation (CS). General (GS) and plastics surgeons (PS) are trained to perform this procedure. Differences in patient population and clinical outcomes between specialties are unknown.

Methods

Using a national database, patients who underwent incisional/ventral hernia repair managed with CS were grouped according to the primary specialty. Patient demographics, perioperative details, and postoperative complications were compared, and the risk factors associated with clinical outcomes were analyzed.

Results

A total of 4,088 patients were identified. PS operated more often in the inpatient setting, and patients had a higher prevalence of hypertension and clean-contaminated wounds. Hypertension and being operated by a PS were associated with an increased risk of needing a blood transfusion after CST.

Conclusion

CS surgical outcomes are similar and comparable specialties. Primary specialty does not affect postoperative complications or 30-day mortality after CS.

## Introduction

Despite the increasing popularity of laparoscopy and robotics in abdominal surgery, open exploration remains a favorable strategy for many general surgeons and surgical subspecialists. The rate of herniation following midline laparotomy incisions has been noted in past studies in up to 26% of cases, with each subsequent abdominal procedure increasing this rate [1-11]. As patient demographics in the United States trend older with increasing rates of obesity, the co-morbidity profile of surgical candidates will also increase [5-6,8-11]. Over the past half-century, complex abdominal wall reconstruction (AWR) as a field has evolved with the continued advent of new technology and various mesh options for the modern surgeon to choose from. Both general surgeons and plastic surgeons tackle the challenge of AWR.

A significant adjunct to addressing difficult AWR has been the use of the component separation technique (CST). First described by Ramirez in 1990 as an anterior approach with incisions made just lateral to the linea semilunaris, it allows surgeons to reduce tension in closures and reconstruct larger defects instead of simply bridging them with mesh. In addition to decreasing tension on the abdominal wall, computed tomography-driven studies have also shown that a 6% increase in intra-abdominal volume can be achieved after a CST repair [2,9]. As concerns over mesh infection rose in the 1990s, CST repair without mesh became increasingly utilized, with a decrease in mesh-related wound complications [9]. Unfortunately, using only CST and forgoing mesh has tradeoffs. Deerenberg et al. evaluated 219 incisional hernias done in this manner. In the review, with the creation of extensive subcutaneous skin flaps from the lateral dissections needed, postoperative complications occurred in ~50% of cases, including complications such as infection, skin necrosis, hematoma, and seroma formation [4]. Studies by Tong et al. and Eriksson et al. noted improved outcomes by combining mesh placement with tension-reducing procedures, such as CST, which reduced the recurrence rates of incisional hernias compared to CST-only cohorts [3,5]. After further prospective studies, systematic data reviews of ventral hernia management recommended that the use of CST in an isolated fashion without mesh support was not advisable [8].

Following the initial introduction of the original technique, CST was then augmented and adapted by surgeons worldwide with various modifications and mesh placement incorporations. These include a distinction between the open anterior (which involves the isolation and division of the external oblique muscle) and the posterior approach (which involves the isolation and division of the transversus abdominis muscle). In an effort to minimize the burden and complications associated with large skin and subcutaneous flap development, endoscopic variants of the posterior and anterior CST have also been developed, as well as a minimally invasive anterior open approach using small lateral incisions separate from the primary laparotomy. Finally, the most recent innovation to be used in a subset of patients has been the introduction of laparoscopic and robot-assisted transversus abdominis release (TAR).

With so many techniques and a lack of individualized current procedural terminology (CPT) coding for each approach to be used in comparative studies, the ideal repair is still a matter of debate. In addition to differences in technique, another unanswered question is whether surgical specialty impacts outcomes in patients requiring an open AWR approach with component separation techniques. In this study, we evaluate the outcomes of CST performed by general surgeons and plastic surgeons by analyzing both clinical outcomes and preoperative risk factors associated with patients undergoing open CST.

## Materials and methods

Database

Patients who underwent incisional/ventral hernia repair and were managed with component separation were identified using the American College of Surgeons-National Surgical Quality Improvement Program (ACS-NSQIP) database.

This database collects over 135 clinical variables, including but not limited to, demographic information, preoperative risk factors, intraoperative variables, and 30-day postoperative mortality and morbidity of patients undergoing major surgical procedures in 11 different surgical specialties [12].

Patient identification

A retrospective analysis of patients who underwent incisional/ventral hernia repair managed with component separation was identified using the ACS-NSQIP participant use data file (PUF) database from 2013 to 2017. Current procedural terminology (CPT) codes for incisional/ventral hernia repair (CPT codes: 49560, 49561, 49565, 49566) and concurrent CPT codes for component separation procedure (CPT code: 15734) were used for this purpose. Patients that underwent mesh implantation (CPT code: 49568), a common procedure performed when repairing ventral hernias, were also included in our analysis.

Patients were further divided according to the surgical specialty that performed the surgical procedure as follows: (1) cases performed by general surgery and (2) cases performed by plastic surgery.

Patients’ demographics, medical comorbidities, American Society of Anesthesiologists (ASA) classification, preoperative laboratory values, perioperative variables, and postoperative complications were compared between groups. An analysis to identify the risk factors associated with postoperative 30-day complications was also conducted.

Statistical analysis

Mean and standard deviation was used to describe normally distributed continuous variables and median and interquartile range to describe non-normally distributed continuous variables. Percentages were used to describe categorical variables. When comparing categorical variables between groups, univariate analyses were conducted using chi-square and Fisher’s exact tests, whereas student t-test and Mann-Whitney U tests were used when comparing continuous variables. Multivariate analyses were conducted to further compare postoperative complications between groups and to identify risk factors associated with postoperative complications. All analyses were performed using SPSS software (2017 IBM SPSS Statistics for Windows, Version 25.0; IBM Corp, Armonk, NY).

## Results

A total of 4,088 cases were identified during the five-year study period. General surgeons performed 3,915 (95.7%) cases; the other 173 cases (4.3%) were performed by plastic surgeons. Mean patient age for the general surgery and plastic surgery groups were 57.7 and 56.1 years, respectively (p=0.098); median body mass index (BMI) was 32 Kg/M2 for the general surgery group and 31.0 Kg/M2 for the plastic surgery group (p=0.157). Plastic surgeons operated more often in the inpatient setting. Patients in the plastic surgery group had a higher prevalence of hypertension. No significant differences were noted in regard to gender distribution, race, smoking history, other medical comorbidities, and ASA classification status (Table 1).

**Table 1:** Patient Demographics SD: Standard Deviation, IQR: Inter Quartile Range DM: Diabetes Mellitus COPD: Chronic Obstructive Pulmonary Disease, CHF: Congestive Heart Failure, HTN: Hypertension, ASA Classification: American Society of Anesthesiology Classification

Variables		General Surgery (%) (n=3,915)	Plastic Surgery (%) (n=173)	p-value
Gender				0.924
	Male	1757 (44.9)	77 (44.5)	
	Female	2158 (55.1)	96 (55.5)	
Race				0.834
	White	3,299 (84.3)	151 (87.3)	
	African American	399 (10.2)	17 (9.8)	
	Asian	14 (0.4)	0 (0.0)	
	American Indian or Alaska	15 (0.4)	0 (0.0)	
	Native Hawaiian	2 (0.1)	0 (0.0)	
	Unknown	186 (4.8)	5 (2.9)	
Hispanic ethnicity		209 (5.3)	10(5.8)	0.864
Mean Age, range, ±SD		57.7 (18 - 89) ±12.8	56.12 (28 - 80) ±11.7	0.098
Median BMI, range, IQR		32.2 (10.7 - 55.1) [9.6]	31.05 (19.6 - 54.8) [9.1]	0.157
Patient care				0.002*
	Inpatient	3,367 (86.1)	163 (94.2)	
	Outpatient	543 (13.8)	10 (5.8)	
	Unknown	5 (0.1)	0 (0.0)	
DM		751 (19.2)	37 (21.4)	0.750
Smoking		712 (18.2)	22 (12.7)	0.066
COPD		255 (6.5)	8 (4.6)	0.322
CHF		20 (0.5)	1 (0.6)	0.598
HTN		2,148 (54.9)	80 (46.2)	0.025*
Dialysis		28 (0.7)	0 (0.0)	0.630
Bleeding disorders		134 (3.4)	8 (4.6)	0.400
Systemic sepsis		52 (1.4)	1 (0.6)	0.688
ASA Classification				0.473
	I	81 (2.1)	4 (2.3)	
	II	1,573 (40.2)	67 (38.7)	
	III	2,151 (54.9)	101 (58.4)	
	IV	109 (2.8)	1 (0.6)	
	Unknown	1 (0.01)	0 (0.0)	

There was no difference in the preoperative BUN, albumin, creatinine, and INR levels between the two groups (Table 2). 

**Table 2:** Preoperative Laboratory values BUN: Blood Urea Nitrogen, HTC: Hematocrit, INR: International Normalized Ratio, IQR: Interquartile Range

Variables	General Surgery (%) (n=3,915)	Plastic Surgery (%) (n=173)	p-value
Median Preoperative BUN, Range, IQR	15 (1 - 102) [7]	15 (7 - 38) [10]	0.775
Median Preoperative Albumin, Range, IQR	4.1 (1.6 - 7.10) [0.50]	4.05 (2.7 - 5.1) [0.50]	0.769
Median Preoperative Creatinine, Range, IQR	0.89 (0.36 - 14.10) [0.32]	0.90 (0.33 - 2.0) [0.35]	0.487
Median Preoperative HTC, Range, IQR	41 (8.8 - 57.9) [5.8]	41 (28.2 - 58) [4.6]	0.855
Median Preoperative INR, Range, IQR	1.0 (0.80 - 4.14) [0.10]	1.00 (0.80 - 1.60) [0.12]	0.050

Perioperative variables demonstrated that plastic surgeons performed more operations in patients with clean/contaminated and contaminated wounds, 13.9% and 4.0%, compared to 8.5% and 1.8% in the general surgery group (p=0.013 and p=0.04, respectively. The median operative time and median length of hospital stay were higher in the plastic surgery group (Table 3).

**Table 3:** Perioperative variables IQR: Interquartile Range

Variables		General Surgery (%) (n=3,915)	Plastic Surgery (%) (n=173)	p-value
Emergency case		56 (1.4)	1 (0.6)	0.517
Wound classification				0.004*
	Clean	3,469 (88.6)	138 (79.8)	
	Clean/Contaminated	331 (8.5)	24 (13.9)	
	Contaminated	69 (1.8)	7 (4.0)	
	Dirty/Infected	46 (1.2)	4 (2.3)	
Median Operative time, Range, IQR		155 (11 - 897) 106	204 (41 - 664) 96	0.0001*
Median Length of hospital stay, Range, IQR		4 (1 - 80) 3	5 (1 - 28) 3	0.002*

With regards to postoperative complications, univariate and multivariate analysis demonstrated that patients of plastic surgeons had a higher prevalence of blood transfusions within 72 hours after surgery start time. Binary logistic regression analysis showed that hypertension and procedures done by plastic surgeons were the two variables associated with this outcome (Table 4 and Table 5). Surgical site infection (superficial, deep, and organ/space), wound dehiscence, deep venous thrombosis, pulmonary embolism, sepsis, unplanned reoperation, and unplanned readmission were low and comparable between specialties.

**Table 4:** Postoperative complications SSI: Surgical Site Infection, DVT: Deep Vein Thrombosis, PE: Pulmonary Embolism, UTI: Urinary Tract Infection, OR: Operating Room

Variables	Univariate Analysis			Multivariate Analysis		
	General Surgery (%) (n=3,915)	Plastic Surgery (%) (n=173)	p-value	Odds Ratio	95% Confidence Interval	p-value
Superficial SSI	198 (5.1)	5 (2.9)	0.280	1.85	0.74 – 4.62	0.185
Deep SSI	89 (2.3)	3 (1.7)	1.000	0.77	0.20 – 2.97	0.715
Organ/Space SSI	82 (2.1)	3 (1.7)	1.000	0.86	0.23 – 3.12	0.823
Wound dehiscence	30 (0.7)	3 (1.7)	0.162	0.45	0.11 – 1.76	0.255
DVT	30 (0.7)	4 (0.1)	0.076	2.79	0.84 – 9.19	0.092
PE	31 (0.8)	3 (1.7)	0.172	0.52	0.14 – 1.90	0.327
Acute renal	16 (0.4)	1 (0.01)	0.521	0.96	0.11 – 8.40	0.972
UTI	56 (1.4)	1 (0.6)	0.517	2.43	0.32 – 18.04	0.384
Bleeding transfusion	69 (1.8)	11 (6.4)	0.0001*	0.28	0.14 – 0.577	0.001*
Sepsis	84 (2.1)	2 (0.01)	0.575	2.34	0.46 – 11.87	0.305
Septic shock	27 (0.7)	1 (0.6)	1.000	1.79	0.20 – 15.94	0.602
Return to OR	185 (4.7)	10 (5.8)	0.524	1.17	0.28 – 4.89	0.826
Unplanned Reoperation	155 (4.0)	7 (4.0)	0.989	0.59	0.11 – 2.95	2.955
Unplanned Readmission	318 (8.1)	8 (4.6)	0.096	1.61	0.71 – 3.65	0.248

**Table 5:** Variables associated with intraoperative bleeding transfusions

Variables		Odds Ratio	95% confidence interval	p-value
Hypertension		0.56	0.35 – 0.90	0.019*
Wound classification	Clean	2.16	0.51 – 9.21	0.295
	Clean contaminated	1.06	0.23 – 4.92	0.934
	Contaminated	0.53	0.10 – 2.77	0.453
Surgical specialty	Plastic Surgery	3.33	1.71 – 6.50	0.0001*

## Discussion

In this retrospective, observational cohort study, using a large-scale national database, we found that general surgeons performed the majority of component separation cases compared to plastic surgeons, however, clinical outcomes are similar and comparable between specialties.

Cases for the plastic surgery cohort had approximately 33% longer operative times than the general surgeon’s cohort, a more significant risk of blood transfusion within the first 72 hours from surgery, and a higher rate of clean-contaminated/contaminated wounds.

To the best of our knowledge, this is the first study comparing component separation procedures between surgical specialties. Previous studies have addressed component separation outcomes based on specific postoperative complications or comorbidities such as pulmonary embolism, obesity, and sarcopenia, among others. However, in all these studies, no differentiation between surgical specialties was made [7,13-14].

The first important piece of information from this study is that nation-wise, general surgeons perform the majority of component separation cases, with more than 95% of the procedures in our study. Although developed by a plastic surgeon, component separation techniques have been used and modified by general surgeons. As noted by Köckerling and colleagues, the increasing complexity of abdominal wall surgery has caused general surgeons to utilize a tailored approach that takes into account the individual patient’s clinical circumstances. Consequently, general surgeons should have adequate training and experience in multiple procedures when approaching an abdominal wall defect, including open and laparo-endoscopic techniques such as intraperitoneal onlay mesh (IPOM), open sublay, open onlay, and open or endoscopic component separation techniques [15]. Given the adjunct benefit of CST in closing larger defects, it is, therefore, a critical tool in a general surgeon’s armamentarium.

In line with this finding, in 2017, Reid et al. utilized NSQIP data from 2007 to 2013 to compare open ventral hernia repair outcomes between general surgeons and plastic surgeons. They found that 99.1% of the open ventral hernia repair cases were performed by general surgeons and only 0.9% were performed by plastic surgeons. Importantly, of the 53,746 patients included in their study, only 2,942 (5%) of them underwent a component separation procedure and no direct outcomes comparison was conducted in this patient population between surgical specialties [16].

Moreover, in our study, cases for the plastic surgery cohort had approximately 33% longer operative time than the general surgery cohort, a more significant risk of blood transfusion within the first 72 hours from surgery, and a higher rate of clean-contaminated/contaminated wounds. This may suggest that plastic surgeons are involved in more complex cases.

Reid et al. found that plastic surgeons usually are involved in complex cases (meaning a higher percentage of component separation cases with an additional panniculectomy, as a surrogate for complexity) with longer length of hospital stay and increased likelihood of contaminated wounds [16]. Similarly, in a published abstract in 2019, a single institution study comparing long-term outcomes of ventral hernia repairs performed by plastic and general surgeons also found that plastic surgeons are involved in more complex procedures than general surgeons (based on the fascial defect area), nonetheless, there were no differences in complications rates between specialties. Importantly, this abstract did not analyze component separation outcomes between surgeon specialties [17].

It is expected that plastic surgeons are involved in complex open ventral repairs, usually in cases such as those with high BMI patients, but it is not uncommon to find collaborations between general surgeons and plastic surgeons. As noted by Chang et al., in a case series of 30 morbidly obese patients who underwent ventral hernia repairs using component separation techniques, the general surgeon performed the operative exposure, lysis of adhesions, take down of enterocutaneous fistulas, bowel resection, and exposure of the abdominal wall, whereas the plastic surgeon will repair the hernia according to the separation of components and the rectus advancement technique [18].

Limitations 

This study is not without limitations; a retrospective review of a national database is always subject to data entry errors and misinterpretation. Specifically, for this study, accurate comparative analysis of the component separation techniques is difficult when all techniques of open CST are grouped into a single CPT code. Therefore, the differentiation of outcomes based upon exact open techniques was not possible. We were not able to differentiate between cases where only a single-sided component separation was conducted versus both sides, which may have shed light on outcomes in patients with concurrent ostomy sites. Furthermore, we did not differentiate results between laparoscopic/robotic CST and open CST codes in this specific study, which could have shown further variability in outcomes, especially with regard to aspects such as hospital stay and pain postoperatively.

Importantly, our study also does not control for variables such as surgeon experience and surgeon patient volume. In regard to training, the background of the plastic surgeon potentially comes into play with AWR, with no ability to easily differentiate plastic surgeons that went through the traditional/independent pathway versus the more recently prevalent integrated pathway of training. Without the experience of a full general surgery residency background (seen in most traditional/independent pathway-trained plastic surgeons), integrated trained plastic surgeons potentially may take longer with AWR cases if they need to involve another surgeon for assistance. This could relate to the assistant lysing intra-abdominal adhesions or performing bowel/fistula resections, instead of having a single surgeon perform the entire procedure without team transitions. The added time seen with these events could be a confounding variable to our results. Also, general surgeons perform a vast amount of surgeries in comparison to plastic surgeons.

We were also not able to differentiate cases based on what institutions they may have been completed in, with well-established hernia centers unable to be separated from the larger data pool. Such centers may have better clinical outcomes that could not be identified.

Moreover, in our study, we were unable to stratify patients by defect characteristics (single/multiple/involving ostomy) or by the sheer total size of the defect given NSQIP data point options. Finally, the limited NSQIP 30-day follow-up timeline will only capture acute complications in open ventral repair. Therefore, long-term complications are not captured by our analysis.

## Conclusions

Open component separation surgical outcomes are similar and comparable between plastic and general surgeons in the acute 30-day timeline. Plastic surgeons are usually involved in more complex procedures; however, this does not translate into higher postoperative complications or mortality after open CS procedures. Further studies comparing laparoscopic/robotic and minimally invasive open and traditional open CST differentiated by the subspecialty of surgeon still need to be completed. Differentiation of AWR results with regard to plastic surgeon training pathway should also be evaluated in the future to see if any variance is seen.
